# Error estimates in atom coordinates and B factors in macromolecular crystallography^☆^

**DOI:** 10.1016/j.crstbi.2023.100111

**Published:** 2023-11-15

**Authors:** John R. Helliwell

**Affiliations:** Department of Chemistry, University of Manchester, M13 9PL, UK

**Keywords:** Coordinates, Atomic displacement parameters, B factors, Precision estimates

## Abstract

The overall diffraction precision index (DPI) of a biological macromolecule crystal structure was first described by Cruickshank in 1999. This topical review proceeds from this point and describes the subsequent elaboration of the index to individual atom coordinates. Additional developments were introduced by the availability of a webserver, which provides a transformed PDB entry with individual atom coordinate errors derived from applying the DPI method using the parameters provided by the authors and then subsequently added to the PDB file. This webserver has been extensively used and harnessed in describing non-covalent distance error estimates as well as assessing the significance, or otherwise, of atom movements in a variety of studies. The standard uncertainties on a biological macromolecule's atomic displacement parameters (the ‘B factors’) has been an entirely different challenge but is obviously important since the crystallographic community has developed the habit of quoting B factors to a false precision in papers. This can convey a false certainty in the dynamics of a structure. A method involving parallelisation of workflows for diffraction image data processing does however offer estimates of the precision of B factors.

## Synopsis

A topical review is presented of atom coordinates and atomic displacement parameters (i.e. the ‘B factors’) and the precision estimates associated with them in a biological macromolecule crystal structure.

## 1 Introduction

In his article, Cruickshank (1999) stated that:

“*reliable σ(x) values are needed for any discussion of non-dictionary distances between atoms in different residues, between protein and solvent atoms or between metal atoms and their ligands.”*Cruickshank (1999, section 10.3)

He derived an equation (Eq. 1 here) to provide an overall diffraction precision estimate of an average atom coordinate error in a biological macromolecule crystal structure, with an average B factor i.e. B_avg_:

(Eq. 1) $$\sigma \left(x,B_{\text{avg}}\right)=1.0{\left(N_{i}/p\right)}^{1/2}C^{-1/3}{Rd}_{\min}$$

Here, *p* = (n_obs_ - n_params_), *R* is the usual residual ∑|ΔF|/∑|F| and *N*_*i*_ is the number of atoms of type i. *C* is the diffraction data completeness, now increasingly very close to 1.0. *d*_min_ is the diffraction resolution quoted by the depositing authors for the respective study under question, at the PDB. This parameter (*d*_min_), is subject to rather arbitrary values, but which has been put on a firm footing by Diederichs and Karplus (2013) who evaluated when the R_free_ (Brunger (1992)) starts to deteriorate upon adding ever higher resolution (i.e weaker) diffraction data. Another important role of Rfree in the current context, instead of R, allowed replacing (n_obs_ - n_params_) with n_obs_ alone (Cruickshank (1999) in sections 6.3 and 7.3) where the number of observations dips below the number of model refinement parameters. This situation could occur at the lower diffraction resolutions (∼3 Å or worse).

Cruickshank (1999) called Eq. (1), the Diffraction Precision Index ‘DPI’ and noted that “*this treatment offered scope for making individual error estimates for atoms of different B and atomic scattering factor”*. Clearly the widely varying B factor values from as a low as 5 to more than 80 Å^2^ in a biological macromolecule crystal structure has by far the biggest effect, since carbon, nitrogen and oxygen have very similar atomic numbers, with sulphur (or phosphorus in nucleic acids) being the exceptions.

**Fig. 1:**
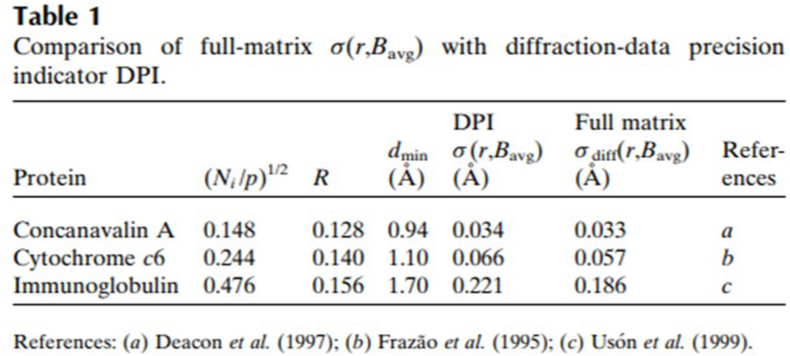
A screenshot of Cruickshank's (1999) Table 1. References: Deacon et al., 1997, FrazaÄo et al., 1995, Uson et al., 1999. Reproduced with the permission of IUCr Journals.

Cruickshank (1999) focussed on the “position errors” σ(r, B_avg_) which are 3^1/2^ times bigger than σ(x, B_avg_), to which we can add σ(y, B_avg_) or σ(z, B_avg_). These three along x, y or z axis will be identical for an isotropic diffracting crystal. A very good confidence in his DPI was established by Cruickshank (1999) by his cross checking against the full matrix inversion estimates (see an example in Deacon et al. (1997) for details of a protein, concanavalin A featured by Cruickshank (1999)). Table 1 from the 1999 paper is reproduced here again as Fig. 1. The practical aspects of the use of the full matrix to determine the errors of atomic positions and temperature coefficients in macromolecular structures, such as its size as well as the ratio of observations to parameters, and the influence of geometric constraints is discussed in detail by Cruickshank (1999). Sheldrick (2008) elaborated on this in his section 2.6 which describes the use of SHELXL for macromolecular refinement for resolutions better than 2 Å including least-squares estimation of individual standard uncertainties.

Gurusaran et al. (2014) encouraged by the implications of Cruickshank, made the necessary extension to individual atoms, with their equation (Eq. 2 here), and thus allowing individuals to calculate the precision found on each atomic coordinate for biological macromolecules, taking into account the B factor of an individual atom versus that of an average atom:

(Eq. 2) $$coordinate\;error\;of\;atom=DPI{\left(\frac{B_{\mathrm{atom}}}{B_{\mathrm{average}}}\right)}^{1/2}$$

Additionally, Kumar et al. (2015) introduced a webserver, which provides a transformed PDB entry with individual atom coordinate errors derived from applying the DPI method using the parameters provided by the authors. This webserver has been extensively used and harnessed in describing non-covalent distance error estimates as well as assessing the significance, or otherwise, of atom movements in a variety of studies.

In an interesting spin off, Blow (2002) developed Cruickshank's (1999) formulae to bring out the dependence of coordinate precision on parameters which are under the experimenter's control in a macromolecular structure analysis, most importantly the resolution. Like Kumar et al.’s (2015) weblink calculator, Blow's (2002) reformulation has encouraged a wider recognition and use of Cruickshank (1999) DPI.

## 2 Precision of a biological macromolecule's atomic displacement parameters

**Fig. 2:**
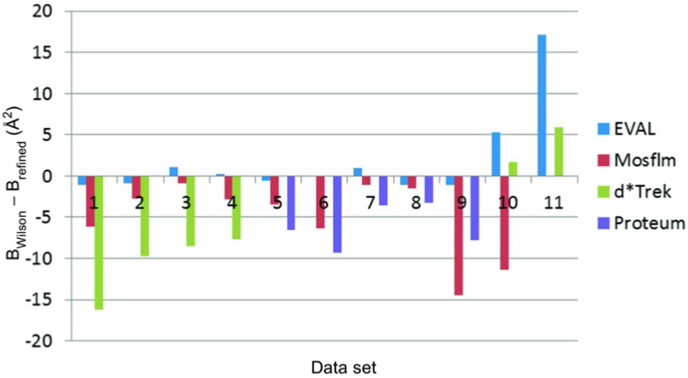
(B_Wilson_ − B_refined_) (Å^2^) for the 11 hen egg white lysozyme crystals studied by Tanley et al. (2013) and the different diffraction image processing software labelled in the graph. 1: 4dd0; 2: 4dd2; 3: 4dd3; 4: 4dd9; 5: 4dd1; 6: 4dd4; 7: 4dd6; 8: 4dd7; 9: 4ddc; 10: 4dda; 11: 4ddb.

To obtain the standard uncertainties on a biological macromolecule's atomic displacement parameters (the ‘B factors’) has been a different challenge to obtaining the standard uncertainties of the atomic coordinates in that there has not been an analytic attempt to estimate their precision. But it is obviously important and needed since there is a current widespread practice in papers quoting B factors with a false precision, even to two decimal places. This can convey a false certainty in the dynamics of a structure. A method involving parallelisation of workflows for diffraction image data processing can be applied and uses a combination of softwares (Tanley et al. (2013)). This methodology offers estimates into the precision estimates of B factors (see their Figure 8 reproduced here as Fig. 2). These comparisons illustrate clearly that B factors have a precision in the range of integers. The diffraction resolutions in the Tanley et al. (2013) studies were 1.7 Å to 2.3 Å. This method has a potential to allow estimates of a spread of the B factor estimates of individual atoms because synchrotron radiation facilities now offer diffraction data processing pipelines with the same diffraction images from one crystal sample processed by several softwares. Specifically, what can be done is that following the first solution of a new biological macromolecular structure, a group of model refinements could be obtained by taking each software's processed diffraction data and re-refine that first model. Comparing these would allow for the calculation of a B factor spread for each atom from those several models. This spread of values, easily converted to an estimated standard uncertainty, would ideally take advantage of as many raw diffraction data processing softwares as possible. Tanley et al. (2013) used Mosflm (Leslie (1999), Battye et al. (2011)), EVAL (Schreurs et al. (2010)), d*trek (Pflugrath (1999)) and Proteum (Bruker (2006)) due to the authors' local preferences. These could be added to by such as XDS (Kabsch (2010)) and hkl3000 (Minor et al. (2006)), which are popularly used.

## 3 Dynamic studies

There are at least two categories of structural change as seen in a single crystal structure study: Firstly, the movement of atoms from one place to another. An example of this method can be of multiple static biological crystal structures to reveal structural dynamics within that population (Tanley et al. (2012)) or under a stimulus such as a laser light flash of a photosensitive molecular system. Secondly, the appearance or disappearance of electron density peaks as a crystal's unit cells show more, or less, spatial coherence - again under some sort of stimulus. As an example of a diffusion stimulus, the appearance of a growing rod of electron density was seen at the catalysis site of the enzyme hydroxymethylbilane synthase as substrate was fed to a crystal in a flow cell (Helliwell et al. (1998)). The first category of structural dynamics requires estimated distance of movement errors and the second category of these requires estimates of electron density differences of putative signal above noise. The latter are usually offered and involve omitting the atoms to give omit electron density maps. However, for the former, uncertainties on the distances which the atoms have reportedly moved, are very rarely (if ever) provided.

The challenge in trying to accurately extract meaningful results where the atom movements are small has been considered in detail by DePristo et al. (2004) who pointed out that “*analyses that depend on small differences in the relative positions of atoms may be flawed*”. Depristo et al. (2004) also quote a Cruickshank DPI coordinate error in their Table 1 of three different protein crystal structures that they highlight.

With the investment in X-ray laser and synchrotron radiation facilities in general and for dynamic crystallography, which often incorporates the study of smaller movements on ever faster timescales, it is a major gap if uncertainties on distances and movements derived from individual atom coordinate errors are not provided. This is one reason why I have also championed the availability of the raw diffraction data underpinning such published studies, as the ‘ground truth’, to be made available directly from the relevant facility via a referenced digital object identifier (doi). The raw diffraction data can then, as a minimum, be checked with multiple processing workflows to evaluate the sensitivity of the results to the various choices made by the original investigators. Likewise different biological macromolecule model refinement workflows can be evaluated too. The routine availability of raw diffraction data across all of macromolecular crystallography, incorporating both novel structures and new methods, is identified as a priority by the IUCr Commission of Biological Macromolecules led by Wladek Minor and now adopted by the IUCr Journals (Helliwell et al. (2019)).

## 4 What does chemical crystallography tell us about the precision and accuracy of its crystal structures?

A statistical analysis of 100 pairs of crystal structures retrieved from the Cambridge Structural Database was reported by Taylor and Kennard (1986). Each structure had been determined independently by two different research groups. The authors remarked in the opening of their paper that:

“*Error estimates are ubiquitous in crystallography; almost all published atomic coordinates and temperature factors are accompanied by estimated standard deviations (e.s.d.'s), which purport to represent the precision of the crystallographic parameters*.”

This happy situation in chemical crystallography arises because a sufficiently high diffraction resolution is nearly always obtained and allows for a full matrix inversion calculation of coordinates and atomic displacement parameters and their *e.s.d.'s* (Taylor and Kennard (1986) cite Rollett (1970)).

Taylor and Kennard (1986) nicely set out the overall nature of the challenge as follows:

“*The value of a particular atomic parameter (p) determined in a particular diffraction experiment may be expressed as: p = μ + ε*_*r*_ + *ε*_*s*_. *Here, μ is the true (unknown) value of the parameter*. *ε*_*r*_*is the 'random error' in the measurement, reflected in the inability of the refined model to fit, exactly, the observed data*. *ε*_*s*_*is the 'systematic error' which arises if the atomic parameter refines to a biased value in order to accommodate certain types of errors in the observations (e.g. absorption)*.”

The first finding which Taylor and Kennard reported was that:

“*The e.s.d.'s of non-hydrogen-atom positional parameters are almost invariably too small. Typically, they are underestimated (on average) by a factor of 1.4 to 1.45.*“

One pair of structures, of squaric acid, had differences between them that were deemed to be due to a systematic error of either differing sample purity or differing crystal mosaic spreads. That pair were omitted from their further analysis.

So, even in the more easily definable and much happier situation of chemical crystallography, cases of small differences in atomic positions between two structures need cautious interpretation since the esds themselves can be slightly biased.

Cruickshank (1999) also reviewed two papers that considered pairs of protein crystal structures and their respective precisions (see Chambers and Stroud (1979) and Daopin et al. (1994)). He found that the two studies made similar comments and that overall, the dominating factor on the precision of atomic coordinates was their atomic displacement parameters.

## 5 Previous attempts

Previous attempts have been made to describe the error estimates in atomic coordinates and B factors within the field of macromolecular crystallography, such as indicated by Kuriyan et al. (1987). Their study was entitled “*Estimation of Uncertainties in X-Ray Refinement Results by Use of Perturbed Structures”.* This study has been cited only 23 times, according to Crossref. Their study was undoubtedly limited by the compute power of the time, at best a Cray supercomputer, and to which the authors deferred, with its limited access.

Another approach is that of using one processed diffraction dataset and assessing ensembles of protein models that are fit to that diffraction dataset. Adams et al. (2013) reviewed these approaches. In their own study, clearly exemplary of its kind, they “*Note that the multimodel representation of uncertainties differs in a fundamental way from individual estimates of coordinate error: In the multimodel representation, it can be seen that relationships between atomic positions (e.g., the shape of part of a side chain) can be retained, whereas the coordinates of each atom are uncertain*.”

## 6 Limitations and restrictions for macromolecular crystallography

There are limitations in applying these estimates to macromolecular crystal structures from other laboratories:

(i) Checks must be made that gross errors of atom misidentification, or inclusion or exclusion when unwarranted by the evidence, are not present. Likewise, an incorrect choice of resolution limit should have been avoided. If the raw diffraction data are made available as well, one can check the estimated resolution limit with the Diederichs and Karplus (2013) method.
(ii) Comparisons between crystal structures at widely different temperatures, such as 100 K to room temperature, are difficult to apply because of the variation in atomic displacement parameters between these temperatures. In addition, there are changes in the structure and dynamics on cooling, which we have noted (see Deacon et al., 1997) as well as others (explained from a physical chemistry viewpoint by Halle (2004) and reviewed recently by Fischer (2021)).
(iii) An isotropic B factor is not an ideal physics-based descriptor (see eg Ploscariu et al. (2021)). It spans both temporal and spatial variations in its single atomic displacement parameter. Disorder situations were specifically excluded from Cruickshank's (1999) DPI analysis. Within those situations I note that split occupancy order (often referred to as ‘static disorder’ in chemical crystallography) should however be treatable by the DPI approach.

## 7 Conclusions

Time-resolved methods as snapshots for studying structural dynamics are growing in importance in the whole field of structural biology and macromolecular crystallography in particular. Furthermore, in a static i.e. non time-resolved macromolecular crystal structure study, crystallisation chemical conditions may be far from the operating conditions of the biological macromolecule in the living cell. Hence variations in structure (which may in principle be very small) between a range of those conditions (see eg Kondrashov et al. (2008)) must be assessed and again emphasises the need for precision estimates of the atom coordinates and B factors of each set of conditions. The ensembles of multiple static crystal structures provided in wonderful graphic forms at the wwPDB need to be accompanied by the intrinsic uncertainties of the structures shown.

In terms of basics is the core point that, as the IUCr Working Party on Expression of Uncertainty in Measurement reported (Schwarzenbach et al. (1995)), “*a measurement result is considered complete only when accompanied by a quantitative statement of its uncertainty*”. The situation of a PDB file not having error estimates on an atom coordinate or on its B factor is a violation of this basic aspect of a measurement result. Today we are not limited in our computing power, unlike Kuriyan et al. (1987) who could make that excuse. This challenge cannot be neglected any longer.

## Declaration of competing interest

The authors declare that they have no known competing financial interests or personal relationships that could have appeared to influence the work reported in this paper.

## Data Availability

No data was used for the research described in the article.
